# LED Light Applied to the Feeder: Impact on Growth Performances of Chickens under Productive Conditions

**DOI:** 10.3390/vetsci10040306

**Published:** 2023-04-21

**Authors:** Livio Galosi, Roberto Falconi, Lucia Biagini, Yulaine Corrales Barrios, Alessandra Roncarati

**Affiliations:** 1 School of Biosciences and Veterinary Medicine, University of Camerino, Viale Circonvallazione 93–95, 62024 Matelica, Italy; roberto.falconi@unicam.it (R.F.); lucia.biagini@unicam.it (L.B.); alessandra.roncarati@unicam.it (A.R.); 2 Department of Morphophysiology, Faculty of Agriculture, University of Camagüey, Carretera de Circunvalación Norte Km 5, Camagüey 74650, Cuba; yulaine.corrales@reduc.edu.cu

**Keywords:** chickens, uniformity, lighted feeder, feeding, growing performances

## Abstract

**Simple Summary:**

One of the major problems in broiler production management is the lack of uniformity in the size of meat chickens. In this study, we assessed the use of feeders equipped with light-emitting diodes and their effects on the productivity of broiler chickens under productive conditions. This particular feeder has been installed at the end of each feeding line to attract broilers and stimulate feed distribution along the entire line. At the end of the cycle, the improvement in uniformity in the poultry house where this equipment was tested, compared to the control poultry house, encouraged the use of this system to improve the production of meat chickens.

**Abstract:**

This study assessed the use of feeders equipped with light-emitting diodes and their effects on the productivity of broiler chickens under productive conditions. A total of 87,200 ROSS 308 chickens, 1-day old, were housed in two poultry houses (CONTROL, F-LED). In CONTROL, 20,000 females (mean body weight 41.12 ± 3 g) and 25,000 males (mean body weight 41.56 ± 3 g) were housed, while 19,200 females and 23,000 males of the same genetic make-up and mean body weight were housed in F-LED under the same environmental conditions. In F-LED, to encourage chickens to feed and to redistribute more feed down the feeding line, a feeder equipped with a LED light has been installed at the end of each line. In CONTROL, no light was located on the feeders. At the end of the cycle, the average body weight never showed significant differences both for females (1345 g in CONTROL; 1359 g in F-LED) and for males (2771 g in CONTROL; 2793 g in F-LED). Uniformity improved in F-LED, at 75.2% in females and 54.1% in males, compared to CONTROL, at 65.7% and 48.5%, respectively, for females and males. The feed conversion ratio followed the same trend, being more favorable in chickens reared in F-LED (1.567) compared to those raised in CONTROL (1.608). The application of a single F-LED at the end of each feeding line demonstrated its utility in improving size uniformity and feed conversion.

## 1. Introduction

In 2021, the world production of poultry meat confirmed a slightly increasing trend, recording a +1.1% increase and reaching 135 million tonnes, thanks to the driving force of countries such as Brazil, China, India, Pakistan, and Mexico, partially offset by slight decreases in the European Union and Indonesia [1]. The European poultry meat sector showed a production of around 13.2 million tonnes [2], of which 1,374,100 tonnes were obtained in Italy. In this country, the poultry meat sector is the only one that is completely self-sufficient in the context of Italian meats: national production, in fact, covers 103.6% of chicken meat consumed [3]. This quota is obtained by applying different systems, mostly identified as intensive poultry production (95%) and a small portion (5%) belonging to an extensive rearing system (organic, free-range, and low-input production systems) [4].

To achieve these important targets, it is known that breeding and management techniques of chickens are key elements to improving productivity [5]. The quantity of feed provided, the nutrient density, the frequency and timing of feed delivery, stocking density, feeder design, and feeder space all affect feed distribution and therefore body weight uniformity [6]. The focus on improving the methods of feed administration is very important since even small decreases in feed intake can lead to significant losses in productivity [7,8]. In the last 50 years, chickens body weight increased by over 450% [9], but in commercial flocks, it is becoming more and more difficult to distribute the right amount of feed to each individual bird.

The ideal poultry house should have dimensions of 150–180 m in length, a width of 15–20 m, and a height of 2.7–3 m. In a shelter of this size, the researchers have shown that the cooling/heating costs are lower than in a smaller shed, thanks to better heat distribution efficiency [10]. Furthermore, in a farm with these characteristics, it is easier to generate a stable microclimate, and the animals will tend to distribute themselves uniformly, occupying all the available space. According to this study [10], a broiler produces about 7 times the heat produced by a man and a layer about 5.5 times, while the consumption of water and feed has grown significantly in the last fifty years. Therefore, with the current hybrids, it is unthinkable to breed with natural ventilation systems. Lighting is an essential component of successful commercial chicken production, able to stimulate and control feed intake [11]. Photoperiod and light intensity are the key elements to maximize growth performances suitable for welfare status [12]. In an overview of the main systems used for rearing commercial broiler and turkeys [13], the use of dim lighting for both meat birds, high stocking densities for broiler chickens and turkeys, and the potential for mechanical failure in automated housing systems are discussed [14]. Most of these concerns persist, and others have emerged as commercial production systems have evolved and public interest in animal welfare has continued to intensify, particularly in developed countries. Light management plays an important role in the growth and behavior of broiler chickens. In the early post-hatching stage, constant lighting has been a common practice in the broiler industry for improving growth performance. However, the effects of constant light in the early life stages of broiler chickens have been rarely reported [15]. Lighting programs stimulate voluntary walking behavior for consuming feed/water and taking rest, as well as improving leg health and performance. This can be used to improve broilers’ welfare and performance on a commercial broiler farm [16]. Light intensity has been investigated to show the effects on growth performance and the bone development of chicken broilers. It has been ascertained that the application of lower light intensity at the starter phase might be a management strategy for broiler farming [17]. Inadequate light photoperiod and a poor environment can have adverse effects on poultry production, and meat yields can be compromised. In a study carried out on the combined effects of lower stocking density, litter components, and a photoperiod based on natural daylight hours, it was found that broiler welfare status improved and the occurrence of foot plant dermatitis was reduced [18]. LED lighting, an acronym that means “light-emitting diode”, is an excellent alternative to traditional lighting systems. It is extremely accurate to administer and capable at the same time of improving both the productivity and quality of life of the animals, as well as efficiency and energy savings. The LED has a longer life—greater than ten times that of a traditional lighting system—and a lower consumption rate, on average equal to one third. It has been demonstrated that LED light has positive effects on the productive costs of chicken due to its long lifespan and duration [17]. It is an optoelectronic device having optical properties of some materials semiconductors to produce photons through the phenomenon of spontaneous emission. This originates from the recombination of electron-hole pairs according to the junction diode principle, characterized by the presence in the device of two suitably prepared parts or areas with opposite electrical traits. A recent review [19] analyzed the light characteristics of the source, intensity, and wavelength of LEDs on different poultry species (chicken, duck, goose, and quail), showing that color and wavelength affected productivity. In a review dedicated to the emerging field of precision lighting [20], LEDs are more often used in livestock houses, providing monochromatic, full-spectrum light comparable with natural daylight in contrast to the previously conventional fluorescent lamps. LED lights show beneficial effects due to their longer life span, lower energy consumption, and low maintenance costs. It has also been shown that monochromatic light positively affects productive performance. Artificial lighting may affect birds that have four types of cones in their retina, so birds see colors differently than humans (three types of cones) [20]. The positive effect exerted by different artificial monochromatic lights has been underlined in different papers focused on lights [21], and all agreed about the positive effects of regulating light in the poultry house.

In commercial broiler farming conditions, among the beneficial effects provided by the LED light system, the flock weight uniformity can be improved. In poultry production, high body weight uniformity is considered one of the most important targets to obtain a standardized process. A low degree of uniformity is also considered negative from an economic point of view because the slaughtering process requires uniform flocks in relation to the projected final mean body weight as agreed with the retailer. This zootechnical parameter represents the rate of birds within 10% of the mean body weight, and the variability of this homogeneity can be reported as the coefficient variation of the individual body weights. Flock weight uniformity can be used as an evaluation of the level of uniformity of the flock with regard to body weight during the rearing of broiler chickens. A uniform flock is related to a low coefficient variation of body weight, whereas low flock uniformity may indicate reduced chicken welfare due to either not suitable housing or management problems [22].

The LED lighting system applied to improve the feed administration to chickens is fully part of the recent branch of research identified with the term precision feeding [6,20,23]. The positioning of the light points highly affects the size uniformity, and these are usually placed at 2.0–2.4 m from the floor in ground farming. This topic could be of interest to farmers because it ensures a fast and uniform distribution of feed along the line, resulting in an improvement in the welfare of chickens. Based on the above, the use of a feeder equipped with LED light (Illuminated FLUXX 330 Big Dutchman, Vechta, Germany) and installed at the end of the feeding lines was examined for its effect on the growing performances of meat chickens, including weight, feed efficiency, and body uniformity, and compared with those exhibited by meat chickens of the same genetics raised in another poultry house with the same constructive traits and management but without the use of supplementary LED light on the feeder.

## 2. Materials and Methods

### 2.1. Experimental Design

The trial was carried out in two poultry houses (CONTROL and F-LED) with a floor area of 2150 m^2^ (length 119.45 × 18 m), located on a farm working in meat chicken production in the Marche Region, Italy. From a construction point of view, both buildings had the same equipment. The walls and the roof were made with 50 mm panels sandwiching internal polyurethane. The ventilation technology consisted of air-forced fans with a capacity of 45,000 m^3^/h, installed at the ends of the walls. Tunnel type, with darkened windows with automatic opening, for the heating air generators, with three burners and ten radiant hoods, was adopted. Cooling panels were also installed. Water and feed were administered ad libitum by five lines of nipples each and four lines of feeders (7 cm/bird).

Both the poultry houses were lighted with conventional light systems based on compact fluorescent lights (CFL), which assured a lighting program respecting the directive on the welfare of broilers in intensive breeding (Dir 2007/43/EC implemented in DL 181 of 27 September 2010, in force since 20 November 2010 in Italy) which establishes that the birds must receive at least 6 h of darkness, of which four are administered consecutively.

**Figure 1:**
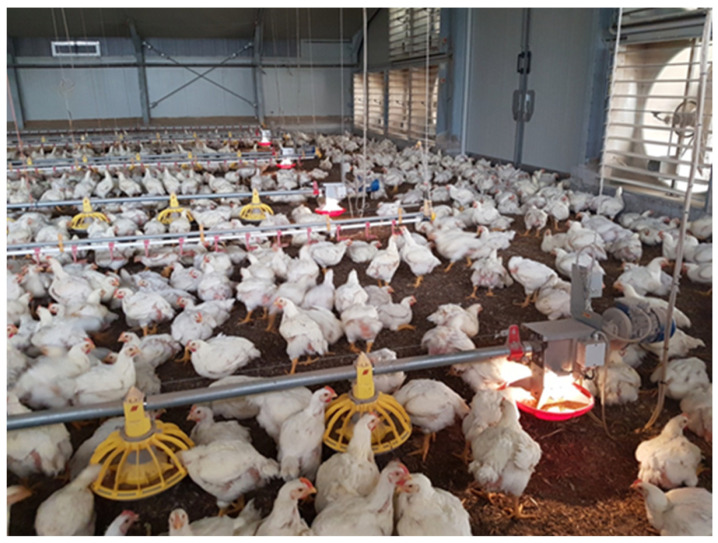
The feeder equipped with a LED light, applied at the end of each feeding line in F-LED.

In F-LED, the CFL lighting system was supplemented by feeders equipped with light-emitting diodes [DC12V (min 10V dc–max 14V dc); 1.2 Watt; light output: 100 lumens; correlated color temperature (CCT) 3000 ± 200K; Illuminated FLUXX 330 Big Dutchman, Vechta, Germany]. This illuminated feeder was located at the end of each line, with the aim to promote feeding and increase feed recall along the entire feeding line (Figure 1). The illuminated feeders were installed before the housing of chickens and were kept on following the general lighting program applied in the building. In CONTROL, no LED light was located on the feeders.

As concerns photoperiod, daily light was 24 h on day 0. Day length was reduced gradually (1 h per day) until reaching 18L:6D on day 7. As regards the light intensity, the recommended range of lux was provided at 30 lux until the end of the cycle. Environmental parameters (temperature, relative humidity) were regulated and monitored by means of a remote sensing system throughout the productive cycle. Each poultry house was divided into two pens by a 40-cm-high transversal net in order to house and rear males and females in separate areas.

### 2.2. Animals

In this trial, a total of 87,200 1-day-old Ross 308 chickens were housed after hatching into CONTROL and F-LED, where both females and males were reared. In CONTROL, 20,000 females (mean body weight: 41.12 ± 3 g) and 25,000 males (mean body weight: 41.56 ± 3 g) were housed, while in F-LED, 19,200 females and 23,000 males of the same genetic background, same mean body weight, and from the same incubator were housed at the same environmental conditions. In both poultry houses, the initial stocking density was around 20 birds/m^2^, both for males and females. All females were captured for slaughter at the age of 29 days, and males utilized the pens of females until 43 days, when they were slaughtered. All birds were fed on an ad libitum basis and received different diets according to the phases of the feeding plan for both groups: crumbled starter feed was initially provided (day 1–day 10), followed by pelleted grower I (day 11–day 21) and grower II (day 22–day 29 for females and day 22–day 36 for males), and then pelleted finisher (day 37–day 43) (Table 1).

During and at the end of the respective growing cycle, 100 females and 100 males were weighed every week in each pen, using an electronic scale (BAT1, VEIT Electronics, Moravany, Czech Republic), provided with data logger software able to calculate the mean body weight and record individual data in a platform used as a database storing historical zootechnical performances of the past productive cycle.

Flock uniformity was expressed by evaluating the body weight of the specimen at the different sampling times and at the end of the growth cycle of females and males in each poultry house and assessing the percentage of birds included within 10% of the mean.

Feed conversion ratio (FCR) was calculated according to the ratio of total feed (kg)/bird (kg). Total mortality was reported, independently by sex, as the sum of the number of dead birds recorded during the daily inspection starting from day 0 to the harvest.

### 2.3. Statistical Analysis

Data concerning body weight, sampled at different times, were subjected to an analysis of variance (ANOVA) using SPSS 25 (Version 25.0, Armonk, NY, USA), and the differences between the means were considered significant at *p* < 0.01 using the SNK (Student-Newman-Keuls) test.

## 3. Results

In Table 2, the microclimate parameters were maintained in ranges suitable to the phase of the productive cycle of chickens in both poultry houses.

Table 3 shows the mean body weight recorded weekly for females and males housed in the two poultry houses. During the trial, the average weight did not show significant differences between the females and the males. At the end of the productive cycle, females reached 1345.43 ± 149.2 g in CONTROL and 1359.42 ± 126.6 g in F-LED, whereas males touched 2771.17 ± 392.7 g and 2793.83 ± 343.7 g in CONTROL and F-LED, respectively. The uniformity rate was improved in the F-LED poultry house where LED lights were applied at the end of the feeding line, showing 75.2% and 54.1% in females and males, respectively, in comparison with the CONTROL, where it was 65.7% and 48.5%. Total mortality recorded a rate that included between 5.57% in CONTROL and 4.56% in F-LED.

**Figure 2:**
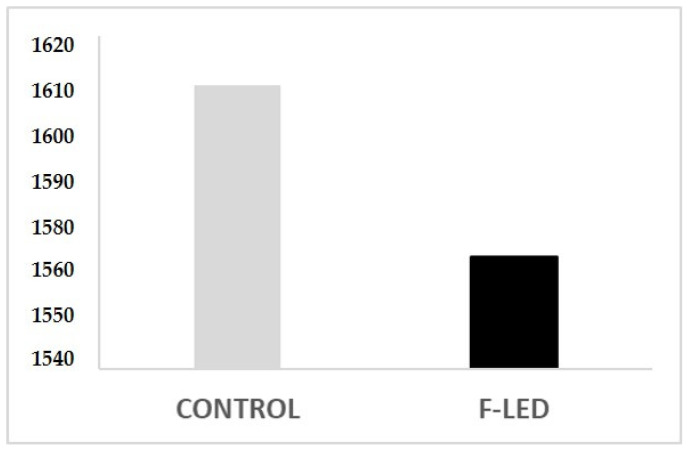
Feed conversion rate of the two groups of chicken obtained at the end of the trial.

Feed conversion rate resulted more favorably in chickens reared in F-LED (1.567) compared to those raised in CONTROL (1.608) (Figure 2).

## 4. Discussion and Conclusions

In the present study, a new generation light source applied to the feeder was considered an interesting system to improve the feed administration and the growing performance of chickens in a productive cycle. According to research performed on the environmental parameters [24], no significant relation was shown between microclimate parameters and body weight uniformity, although indirect consequences can be found.

This cutting-edge technology has been recognized as being able to increase the growth of poultry. In a recent review, a comparative analysis of incandescent lamps, compact fluorescent light (CFL), and emitting diodes (LED) found positives and limitations of these three light sources in poultry farming. LED light emerged as the most promising technology to guarantee high growth performances and save energy costs [19].

This situation was in line with the results of a study on monochromatic LED light evaluating light in broiler houses that might have the potential to improve broiler welfare [25].

Furthermore, the work performed on the light environment in broiler breeder houses with three different light sources (CFL, LED, and UVA) [26] showed that natural light environments were not static. During dusk and dawn, the intensity as well as the spectral composition change gradually, and even during the day, light changes dynamically due to the movements of the sun and changing cloud cover. These continuous changes in the light environment are important for the behavioral control of all animals.

In the current study, the application of a LED light on the feed pan, positioned at the end of every feeder line of the poultry house, was tested in order to evaluate the effects on the growing cycle of chickens compared to those reared without this additional light source. We tested an LED light system having a CCT of 3000 K, considered cool because it contains more blue color than the conventional one (CFL, 2700 K). Diodes CCT were cooler (4500–5300 K) than those tested and reported in literature [19] aimed at examining different productions (eggs). A potential positive effect of neutral LED lighting on animal welfare in terms of biochemical parameters was also shown in a study aimed at evaluating the effects of neutral (K = 3300 to 3700), cool (K = 5500 to 6000), and warm (K = 3000 to 2500) LED lightings. This management strategy could induce positive changes in the fecal microbiota, leading to an improvement in the chickens’ health [27].

Satisfactory results using white LED light on chicks were reported [8], whereas another study reported no effect on body weight [28]. In more recent research, the different white LED light colors increased activity when compared to blue or green light, showing that light color has an impact on the chick’s behavior [29].

The final mean body weight, also without significant differences between F-LED and CONTROL, showed a higher uniformity in body weight in chickens, both females and males, reared with the innovative illuminated feed pans compared to those kept under conventional light. At the base of this condition is a low level of uniformity, which can also show differences in access to feed and water. Comparing the administration of natural light with traditional LED in poultry houses, Linhoss et al. [30] ascertained light levels significantly increased in the environment and the overall spatial uniformity was very low; it is well known that spatial and temporal variability lead to feed differences and unbalanced water consumption, decreased carcass quality due to scratching, and decreased litter quality from excess bird density in some areas of the house. In our case, the uniformity of the F-LED group showed better results than those reported in the literature. Other studies ascertained that a high stocking density (23.8 broilers/m^2^) in comparison with a low bird concentration (11.9 birds/m^2^) gave lower uniformity rates (13.0% and 15.3%, respectively), but both of these cases were significantly lower than that obtained in our current trial [22].

Homogeneous carcasses allow for maximizing the yield at the slaughterhouse, reducing off-range products, and increasing the quality of the cutting and boning phases. In all cases, poor body weight homogeneity reduces the potential for ongoing success because of suboptimal performances both in overweight and underweight birds [31,32].

Thanks to the placement on the last point of the feeder line, only one F-LED lamp was used in each line. Probably, in this trial, the F-LED allowed a more rapid recall of feed and a better feed distribution across all the feeding lines than in the conventional feeding system. Other studies are needed to confirm the utility of the F-LED in improving uniformity in poultry flocks and the use of the device with different colors and lighting times. Differently, another study needed the LED light application in all the feeding areas and also over the drinking point [33] to obtain results similar to ours concerning final body weight and mortality. In our study, the final mean weight resulted slightly higher than that obtained in that study, where both sexes were reared together and the uniformity size was not investigated. The cumulative mortality (3.5%) is lower than the total death rate (5.2%) in chicks reared in a poultry house with standard lighting. Furthermore, in our trial, total mortality was in line with this range, recording the low mortality in F-LED. Although there were no replicates, chickens in F-LED exhibited more favorable results in terms of feed conversion rate [34,35]. The tested F-LED triggered an important adaptive behavior in the animals, which, learning to recognize the noise of the auger, were stimulated to assume the feed more frequently, as shown by other studies [36]. When the feeding line became empty, the fast recall of feed avoided residuals and thereby assured high feeding hygiene conditions.

The present study demonstrated the potential traits of feeders equipped with LED lights to improve chicken uniformity. LED light is widely employed in commercial poultry farming, but in the current trial, the innovative type of feeder assured an accurate feed supply frequency to chickens along all the feeding systems, thanks to the positioning of only one device at the end of the line. This device can be integrated into the existing line without drastic changes in the equipment available in the farm, guaranteeing savings in the costs of the productive cycle.

## Figures and Tables

**Table 1:** Chemical composition (% as it is) and metabolizable energy calculated (ME) of the feeds administered to chickens in both poultry houses.

	Starter	Grower I	Grower II	Finisher
Protein	22.8	20	19.5	17.9
Lipids	5.5	6.6	8.2	7.6
Fibre	3.2	3.1	3.3	2.9
Ash	6.4	5.4	4.9	4.4
Lysine	1.48	1.29	1.26	1.13
Methionine	0.36	0.31	0.30	0.28
Calcium	0.94	0.77	0.66	0.59
Phosphorus	0.60	0.49	0.42	0.37
Sodium	0.16	0.16	0.16	0.15
ME (kcal/kg)	3010	3175	3180	3225

**Table 2:** Internal temperature and relative humidity recorded in the two poultry houses during the trial.

	Temperature (°C)		Relative Humidity (%)	
	CONTROL	F-LED	CONTROL	F-LED
Day 1	32	32	57	57
Day 5	30.1	30.1	56	56
Day 8	29.3	29.3	59	59
Day 15	26.8	26.8	61	61
Day 22	23	23	62	62
Day 29	21.2	21.2	60	60
Day 36	19	19	62	62
Day 43	17.7	17.7	64	64

**Table 3:** Females and males reared in poultry houses without (CONTROL) and with feeders equipped with light-emitting diodes (F-LED): final body weight (mean ± standard deviation) and flock uniformity.

	CONTROL	F-LED	CONTROL	F-LED
	Mean body weight (g)		Flock uniformity (%)	
*Female*				
**Day 1**	41.12 ± 3.3	41.12 ± 3.3	81.1	81.1
**Day 8**	144.00 ± 15.8	143.08 ± 17.9	62.0	61.2
**Day 15**	405.86 ± 46.9	394.52 ± 49.7	65.7	52.3
**Day 22**	800.55 ± 82.2	814.22 ± 88.3	66.3	64.2
**Day 29**	1345.43 ± 149.2	1359.42 ± 126.6	65.7	75.2
*Male*				
**Day 1**	41.56 ± 3.0	41.19 ± 3.0	85.1	85.1
**Day 8**	145.16 ± 16.4	142.78 ± 16.5	60.7	65.3
**Day 15**	411.73 ± 54.1	416 ± 47.1	59.0	59.2
**Day 22**	854.73 ± 125.3	876.99 ± 105.3	48.5	58.5
**Day 29**	1486.94 ± 171.0	1542.19 ± 169.8	60.5	58.4
**Day 36**	2038.86 ± 248.9	2064.28 ± 228.7	58.5	63.2
**Day 43**	2771.17 ± 392.7	2793.83 ± 343.7	48.5	54.1

## Data Availability

All data are included in the article.
